# Systematic Organization of COVID-19 Data Supported by the Adverse Outcome Pathway Framework

**DOI:** 10.3389/fpubh.2021.638605

**Published:** 2021-05-19

**Authors:** Penny Nymark, Magdalini Sachana, Sofia Batista Leite, Jukka Sund, Catharine E. Krebs, Kristie Sullivan, Stephen Edwards, Laura Viviani, Catherine Willett, Brigitte Landesmann, Clemens Wittwehr

**Affiliations:** ^1^Institute of Environmental Medicine, Karolinska Institute, Stockholm, Sweden; ^2^Environment Health and Safety Division, Environment Directorate, Organisation for Economic Cooperation and Development, Paris, France; ^3^European Commission, Joint Research Centre, Ispra, Italy; ^4^Physicians Committee for Responsible Medicine, Washington, DC, United States; ^5^GenOmics, Bioinformatics, and Translational Research Center, RTI International, Research Triangle Park, NC, United States; ^6^Humane Society International, Washington, DC, United States

**Keywords:** adverse outcome pathways, COVID-19, systematic organization, interdisciplinarity, mechanisms, data integration

## Abstract

Adverse Outcome Pathways (AOP) provide structured frameworks for the systematic organization of research data and knowledge. The AOP framework follows a set of key principles that allow for broad application across diverse disciplines related to human health, including toxicology, pharmacology, virology and medical research. The COVID-19 pandemic engages a great number of scientists world-wide and data is increasing with exponential speed. Diligent data management strategies are employed but approaches for systematically organizing the data-derived information and knowledge are lacking. We believe AOPs can play an important role in improving interpretation and efficient application of scientific understanding of COVID-19. Here, we outline a newly initiated effort, the CIAO project (https://www.ciao-covid.net/), to streamline collaboration between scientists across the world toward development of AOPs for COVID-19, and describe the overarching aims of the effort, as well as the expected outcomes and research support that they will provide.

## Introduction

The coronavirus disease 2019 (COVID-19) pandemic engages a large number of scientists across the world. Many countries have adapted and re-assigned their research funding schemes, and established expert teams engaged in studying and providing information about the outbreak. Literature and data are emerging quickly and have been made openly available to support reuse. A single-word search on “COVID-19” in PubMed generates a result of 63,481 papers published this year (accessed October 15th, 2020). Several life science data management infrastructures have acted quickly to provide a range of services, such as databases, software tools, and cloud storage, for handling and analyzing the data. For example, the European intergovernmental organization ELIXIR supports COVID-19 data management with a wide variety of services (https://elixir-europe.org/services/covid-19), and national branch infrastructures have appeared, including the COVID-19 Data Portal in Sweden (https://covid19dataportal.se/) or the Portuguese inter-institutional initiative (https://colife.eu/en/). In the US, the National COVID Cohort Collaborative (N3C) is coordinating the collection and analysis of clinical, laboratory, and diagnostic data (https://ncats.nih.gov/n3c), including data generated by the National Center for Advancing Translational Sciences (NCATS; https://opendata.ncats.nih.gov/covid19/). Interesting artificial intelligence-driven data compilation initiatives, as well as efforts to harmonize terminology (i.e., ontology developments) are also arising (1); https://github.com/CIDO-ontology/WCO/.

These enormous worldwide joint research efforts on one specific disease provide us with a unique opportunity to map and understand human biology and the response to virus infection in-depth. However, the immense amounts of information and data are difficult to organize, integrate, and evaluate in order to develop a comprehensive understanding of the disease and apply the knowledge in real-world practice. The Adverse Outcome Pathway (AOP) framework provides a much-needed common basis and infrastructure for collaboration across various disciplines. The aim of this Perspective is to provide an overview of the goals in a newly initiated effort to apply the AOP framework for systematic organization of COVID-19 research.

## Why Adverse Outcome Pathways for COVID-19?

The AOP framework was developed to provide a means for interdisciplinary and systematic gathering, organization, and review of highly variable types of available data and information across multiple levels of biological organization. An AOP organizes information pertaining to a biological process describing a sequence of causally connected biological events that lead to and are essential for the rise of an adverse outcome, such as that caused by chemical exposure or disease. One of the main aims of the framework is to provide mechanistic understanding of health effects allowing for development of mechanism-based and increasingly human-relevant (i.e., ideally animal-free) model systems for safety testing, diagnostics and drug development (2). The AOP framework was formalized by the Organisation for Economic Cooperation and Development (OECD) in 2012 (3, 4), when an AOP Development Programme was introduced and the AOP-knowledgebase (AOP-KB), including the AOP-Wiki (https://aopwiki.org/) was established. It has, to date, mainly been applied within the field of toxicology and chemical or nanomaterial risk assessment (5–7), but due to its central conceptual principles and guidelines, it is widely applicable to any medical field associated with the need for elucidating underlying mechanisms of disease (8).

**Figure 1:**
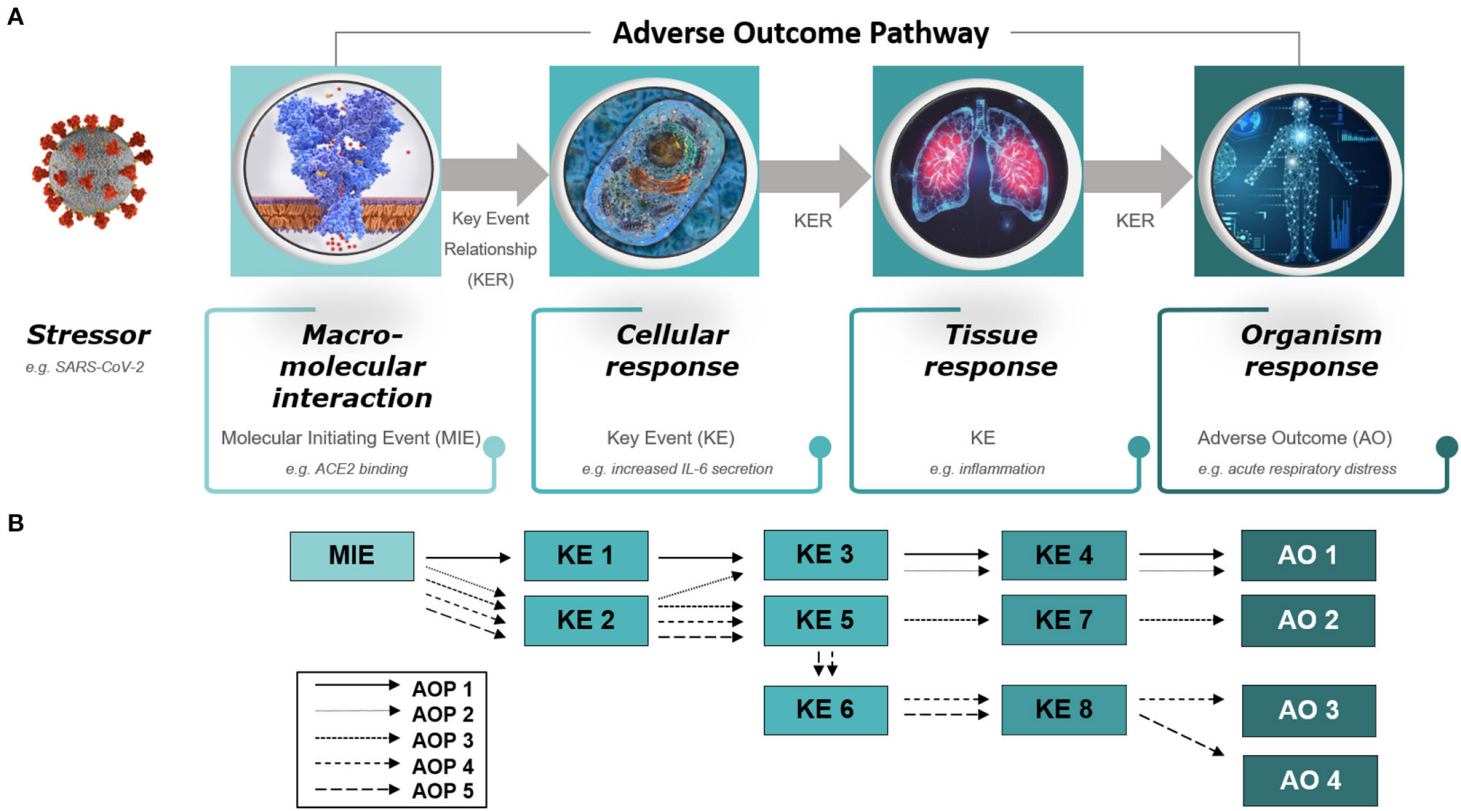
Diagram of **(A)** an adverse outcome pathway (AOP) and **(B)** an AOP network. **(A)** An AOP starts with a molecular initiating event (MIE), followed by a series of key events (KEs) on different levels of biological organization (cellular, tissue, organ) and ends with an adverse outcome (AO) in an organism. The stressor is not part of the AOP itself. The diagram provides examples of an MIE, KEs, and an AO relevant to COVID-19, that is, ACE2 binding leading to IL-6 secretion, inflammation and acute respiratory distress. **(B)** AOP networks depict interrelated AOPs that share KEs, referred to as hub KEs, which, for example, may support identification of mechanisms central to diverse courses of action taken by COVID-19 in different patients. Please refer to reference (9) for a preliminary overview of a detailed AOP network for COVID-19.

In particular, two main principles guiding development of AOPs allow for broad applicability of the framework; the first being the fact that AOPs are stressor independent (8). This means that rather than describing and characterizing the specifics of the stressor and its associated mechanism of action, the AOPs focus on the biology and what happens once the cascade toward disease has been initiated. This inherent property of AOPs makes them useful for describing adverse outcomes associated with any type of stressor, whether it is a toxic substance or a virus. The second crucial principle is the modular structure of AOPs, which are composed of reusable components referred to as key events (KEs) and key event relationships (KERs) (8). KEs and KERs describe the causally connected events taking place at different levels of biological organization, from molecular initiating events (MIEs) to organ-level events, essential for the adverse outcome (AO) to happen (Figure 1A). Thus, the reuse of KE/KER components previously developed and described within other fields of research (and in relation to other types of stressors) may provide relevant information already captured in AOPs, potentially jump-starting efforts toward understanding COVID-19. The modular aspect also allows for the development of so called AOP networks where the complexities of interrelated AOs are brought to justice, and shared hub KEs become evident (Figure 1B) (8). This is particularly interesting for COVID-19 as the disease seems to result in widely diverse types of outcomes, due to yet unidentified influences, modulating factors and predispositions (10). Hub KEs may identify mechanisms central to the diverse courses of action taken by the disease process in different patients [See further detailed description of the principles of AOPs in (8)].

In addition, a third principle of the AOP framework—and perhaps the most significant benefit to COVID-19 research—brings collaboration within science to new levels. AOPs are living documents and should therefore continuously be finetuned and updated as new information becomes available. Thus, development and maintenance of AOPs requires contribution and engagement of scientists from widely diverse fields. Gathering and reviewing of knowledge by different experts constitutes a trustworthy source of information in the midst of a scientific trust crisis (11).

In line with these thoughts, recent efforts have emerged suggesting the application of the AOP framework for improved understanding of COVID-19, including development of two putative AOPs in the AOP-Wiki [AOP 320 and 319, https://aopwiki.org/aops/319; https://aopwiki.org/aops/320; (9, 12)], an exploratory approach to develop an AOP-linked molecular description of COVID-19 [https://www.wikipathways.org/index.php/Pathway:WP4891; (13)], and the publication of a putative AOP for pneumonia related to COVID-19 (14). These initial efforts have focused on the most frequently reported outcomes of COVID-19, that is, effects on the lungs including pneumonia, acute respiratory distress, and fibrosis. As mentioned previously, it is now clear that COVID-19 also affects other organs, including the cardiovascular system, the kidneys, and the nervous system, as well as multi-organ failure due to systemic dysfunction (10, 15, 16). In addition, sex-related differences and chronic effects such as development of fibrotic lesions in the lungs are becoming increasingly evident (17–19). Thus, there is an urgent need to map and organize arising information and data for the benefit of increased understanding of the variable outcomes. In support of this need, many of the outcomes and central mechanisms currently described in relation to COVID-19 can be found in existing AOPs, KEs and KERs developed within the field of toxicology and stored in the AOP-Wiki, including an AOP network describing a set of lung injuries and systemic dysfunction involving the cardiovascular system (5, 20). Other potential support can be found from AOPs describing liver injury, as well as a set of hub KEs covering the inflammatory processes often associated with a multitude of AOs in diverse organs (21, 22).

Here we describe the aims and objectives of the European Commission-initiated project CIAO, which broadly covers the aspects outlined above by “modeling the pathogenesis of COVID-19 using the Adverse Outcome Pathway framework” (www.ciao-covid.net/). The project is coordinated by the European Joint Research Center (JRC) together with partners from Humane Society International, Physicians Committee for Responsible Medicine, the OECD Environmental Health and Safety Division, and scientists from the Karolinska Institute and Research Triangle Institute International, with the overarching goal to provide a structured way of organizing and translating scientific evidence into simplified and trusted messages supporting efficient political actions. The main objectives of the CIAO project are (i) to allow for interdisciplinary collaboration toward better understanding of the disease through establishing a unique overview of the complete COVID-19 disease process(es), (ii) to foster crowdsourcing, and (iii) to establish a workflow and infrastructure for systematic handling of data in potential future health crises.

### Interdisciplinary Collaboration to Gain a Complete Overview of COVID-19

Collaboration is one of the main tools when producing science for regulatory purposes. As stated in the recently published JRC Handbook of Science for Policy, this process is even considered “vital for modern scientific organizations producing relevant input in a policy context.” Such collaboration is preferably done through communities of practice that share a domain of interest and engage in joint activities, discussions, and the practice itself (23).

However, collaboration between scientists from different fields, as well as between scientists and stakeholders of research results (e.g., pharmaceutical companies, clinicians, decision-makers, regulators), is notoriously difficult due to different vocabularies, variable focus on end-use of results, unconnected data management strategies, lack of channels for communication, and a lack of epistemic trust (24). AOPs provide a basis for harmonized communication and collaboration toward common goals and can be seen as providing a simplified “front page” overview of KEs representing “abstracts” of in-depth research, which is maintained within the framework in the form of detailed descriptions, reference to literature and links to curated biological pathway databases (25). Common goals for multi-disciplinary teams can for example be to elucidate KE-related mechanisms, to fully describe KERs, to develop or provide context for KE-targeted alternative model systems and methods for safety testing, diagnostics, and drug development, or to have a scientific basis upon which to take decisions and develop regulation for diverse clinical strategies. Thus, AOP-supported collaborative efforts can be expected to provide a broader overview of COVID-19 and help identify both knowledge ready for real-world application and areas in need of further research, as described in detail in the next section (What can AOPs do for COVID-19 research).

### Foster Crowdsourcing

AOP development is dependent on joint crowdsourcing, especially the continuous need for refinement and updating of existing AOPs in line with new research and data. The AOP-KB, and especially the AOP-Wiki supports and fosters crowdsourcing, giving researchers a basis upon which to target their research and build their projects. The OECD oversight and governance of the AOP framework supports its wide acceptance, including by regulatory agencies, as a basis for collaboration and supports linkage of research results with translational real-world applicability. This has been demonstrated within toxicology, through the linkage of AOPs with OECD Test Guidelines (TGs) used for chemical safety assessment (3). The OECD-supported harmonization and standardization brought to operating procedures, test systems and research consensus in general, provides a broad basis for application in official decision-making and regulation.

Large scale AOP-aligned crowdsourcing has been limited to date, and development of AOPs has often been driven by research efforts dependent on diverse time-limited funding strategies (26). Challenges for implementation of wider community contribution to the framework include, for example, a current lack of scientific acknowledgment for researchers publishing in the AOP-KB, and unclear strategies for maintenance and updating of published AOPs. However, broader sustainability plans for the framework have been discussed and a road map toward a proactive, cohesive, and targeted strategy has been outlined (26). The current pandemic is a unique opportunity to put the road map in action on a much larger scale and can be expected to generate insight into further development needs of the AOP framework itself, as described in detail in the section “What can COVID-19 research do for AOPs.”

### Establish Infrastructure for Potential Future Health Crises

**Figure 2:**
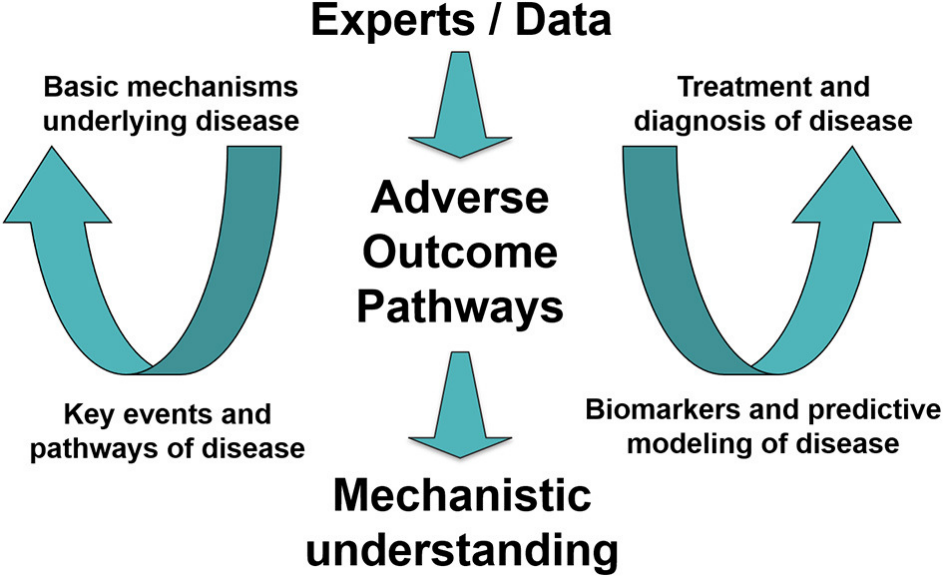
Toward an interdisciplinary workflow and infrastructure for systematic gathering and organization of data in potential future health crises. A concept demonstrating how experts join in an interdisciplinary data-driven effort supported by the Adverse Outcome Pathway (AOP) framework to increase mechanistic understanding of disease. The gained understanding supports effective (and ideally animal-free) development of treatments and diagnostics. [Figure modified from (28)].

Solving human and environmental crises is dependent on interdisciplinarity among a wide variety of actors. Future health crises similar to the ongoing pandemic can be expected to rise again as a consequence of, among other things, climate change, which is linked to an increased risk for communicable diseases and/or the emergence of such diseases in new contexts. Even before the COVID-19 crisis, researchers were pointing to the need for public health providers to be ready to play an active role in prevention, early detection, and mitigation of future health effects associated with climate change (27). In relation to this need, it has also been emphasized that understanding and staying abreast of new strategies for maintaining such an active role will be crucial for preparedness and resilience among scientists and health professionals (27). Preparedness and resilience require the systematic gathering and organization of data and information, which in turn requires communication, collaboration, structured processes, and an infrastructure allowing for the representation of scientific evidence in clear and useful ways. Thus, as described above, AOPs provide a well-established basis for the needed structured approach supporting interdisciplinary synergy among scientists and health professionals enabling resilience and quick action in the future (concept outlined in Figure 2).

## What Can AOPs do for COVID-19 Research?

AOPs do not produce new data, but rather provide a platform, not only for organizing and integrating the abundant available relevant data and information from any related field, but also for interconnecting scientists and experts from various fields. This holistic and collaborative approach facilitates curation of data, discussions, and an improved knowledge exchange between people that work and research on the same problem but in different fields.

In addition, AOPs provide insight into and synthesis of prior knowledge gained within other fields of research, such as toxicology, where the framework has been applied and utilized for the purpose of better understanding chemical- and nanomaterial-associated adverse effects. A wide variety of AOPs covering lung, cardiovascular, liver, and neurological dysfunction, as well as hub KEs describing inflammation and oxidative stress have been developed and all provide a basis and starting point for unraveling effects associated with COVID-19 (5, 21, 29). In addition, systematic processes for cross-disciplinary application of information and AOPs/KEs developed within chemical toxicity research to nanotoxicology have been described and provide further substance for widening the application of AOPs to COVID-19 (30).

Although AOPs may not produce new data *per se*, interlinking existing data and information across biological levels from molecular up to population level may provide insights and new knowledge that are not evident when data are interpreted in isolation. This supports a better understanding of the underlying pathophysiological processes and an improved basis for interpreting and identifying underlying reasons for the various clinical courses of the disease. For example, there may be turning points in the natural history of the disease, which, when identified, might allow for therapeutic intervention prior to clinical manifestation (31). In spite of massive world-wide research into COVID-19, there is still much we do not know and understand. The depiction of available data along the pathway can identify specific knowledge gaps and direct further research. Furthermore, controversial and contradictory findings can be compared and put in relation to known underlying mechanisms for further clarification.

Another benefit of the framework comes from the fact that AOP-supported descriptions of various organ manifestations may result in AOP networks that reveal, not only the interrelation between various outcomes, but also major pathway interconnections and hub KEs that can serve as diagnostic markers or targets for therapeutic interventions. Furthermore, AOP networks help elucidate the role of co-exposure and the ways comorbidities and other modulating factors influence the disease outcome. This aspect is especially supported by knowledge from the field of toxicology where certain universal responses to exogenous exposures are well-described, for example, the previously mentioned process of inflammation (22).

Last but definitely not least, the mechanistic understanding of COVID-19 has a significant potential to support quicker and more human-relevant non-animal-based data generation and new mathematical prediction models for the effective development of preventive, diagnostic, and therapeutic strategies (32).

### Who Will Benefit From COVID-19 AOPs?

The end users of the resulting COVID-19 AOPs and associated organized knowledge are likely to be of widely variable disciplines, analogous to the developers of the AOPs (Table 1). Initially, the primary users could be assumed to be pharmacologists and clinicians seeking aid in development of treatments and diagnostics for COVID-19. In drug development, AOP knowledge can inform efficient (non-animal) model systems and methods for target discovery and safety testing (and eventually perhaps also efficacy and quality testing) (32). In addition, AOP knowledge can be used to guide the planning of new research, synergistic collaboration and cross-fertilization opportunities amongst transdisciplinary scientists.

**Table 1:** Multi-disciplinary range of COVID-19 AOP developers and stakeholders.

	**Developers** ** (give knowledge)**	**Stakeholders** ** (take insight)**
Virologists	Virus function and background	Disease overview
Epidemiologists	Disease spreading	
Immunologists	Role of the immune system	Gap bridging
Biologists	Biological mechanisms	
Computational scientists	Modeling and prediction	Cross-fertilization
Clinicians	Course and outcome of the disease	
Pathologists	Damage assessment	Transdisciplinarity
Pharmacologists	Pharmacological mechanisms	
Toxicologists	Toxicant-induced MIEs/KEs/KERs/AOs	Informed science-based decisions and mitigation measures
Risk assessors/ managers		
Innovators, industry	Specific sectoral knowledge	Prioritized investments and research funding
Decision makers		
Regulators		
Virtually everyone		

*For additional detail, please refer to (26)*.

Due to the simplified nature of the AOPs and their aim to provide overviews and summaries of complex biological processes, their potential impact is much wider than that and reaches all the way up to the level of general communication across risk assessors and managers, innovators, decision makers, and regulators (3). Current knowledge is synthesized through the AOP framework in a meaningful way and allows for weight of evidence to be transparently communicated, supporting science-based regulatory decisions and making science accessible to non-experts. For example, AOPs may support informed mitigation measures and public communication through reference to concordant, convincing and unbiased evidence gathered in AOPs, which in turn aids, among other things, endeavors to counter widespread conspiracy theories about COVID-19 (33). The nature of the AOP-Wiki can be compared to the now widely trusted and crowd sourced encyclopedia, Wikipedia, which over the last few decades has grown from a highly doubted source to one of the main reliable sources of general public information (34). The AOP-Wiki has the benefit of an added layer of transparent evaluation of the underlying information. In addition, the support that AOPs provide for the identification of existing knowledge and elucidation of research gaps can guide prioritization of investment in research funding (26). Overall, AOPs provide scientists with a much-needed basis for joint presentation of research consensus as well as a general disease overview within the rapidly progressing field of COVID-19 research.

## What Can COVID-19 Research do for AOPs?

The support that AOPs provide for COVID-19 research reaches back to the development of the AOP framework itself, as referred to on several occasions above. This synergistic feedback loop is emphasized by the key principles of the framework: AOPs are stressor-agnostic, they are modular, and they are living documents. Thus, the information and understanding gained from unraveling the mechanisms (MIEs, KEs, KERs, and AOs associated with COVID-19) can be assumed to reach back into and inform other fields (e.g., toxicology and chemical safety assessment). In fact, such cross-feed of information between fields, such as cancer research and disease models development, has repeatedly been proven to be useful. For example, big data analytics within toxicology frequently applies data sets originally derived from other fields to develop and test mechanism-driven predictive models (35–38). Once structured and organized to be associated with the modules of AOPs, this type of data becomes broadly informative across disciplines (28).

In addition, the application of the AOP framework to map a disease of high societal relevance will provide valuable experience for the evolution of the framework itself. This effort can be expected to inform potential needs for adaptations of the framework to improve the strategies for its maintenance and sustainability. As stated by Carusi and colleagues, “although the AOP concept has the potential to significantly impact the organization and interpretation of biological information in a variety of disciplines/applications, this promise can only be fully realized through the active engagement of, and input from multiple stakeholders, requiring multi-pronged substantive long-term planning and strategies” (26).

Overall, the application of AOPs to a highly relevant disease such as COVID-19 is a unique case study providing opportunities with far-reaching possibilities, and it allows for broad inclusion and wide-spread dissemination of highly diverse research results, all contributing to the bigger picture.

## Conclusions

Overall, the development of AOPs and AOP networks for COVID-19 can be expected to support a set of at least nine research needs, as identified here, including (i) means for improved knowledge exchange between experts from different disciplines, from virology to clinical research, (ii) increased synthesis of prior knowledge from other fields, such as toxicology and medicine, (iii) increased understanding of the variable courses of the disease, (iv) means for identifying knowledge gaps, (v) means to compare and contrast data and knowledge to better understand controversies and uncertainties, (vi) increased understanding of the interrelation of the various outcomes, (vii) detection of new biomarkers (which have direct evidence of causality and essentiality for the outcome, in line with the principles of the AOP framework), (viii) understanding of the meaning of co-exposure and potential risk factors, and (ix) increased mechanistic understanding of the disease to support human-relevant data generation.

In addition, thanks to today's state-of-the-art technological advances with COVID-19 there has been a never-before-seen tsunami of data focused on one particular human disease. Application of the AOP framework to the systematic organization of this unprecedented amount and breadth of data can be expected to effectively support the evolution and improvement of the framework itself. Thus, in the time of the COVID-19 pandemic, the AOP framework, and in particular the European Commission CIAO project, has the potential to provide a concrete example of how a structured approach based on inter- and multidisciplinary synergy among scientists and health professionals could facilitate data exchange, identify knowledge gaps, redirect funding, and increase success in the understanding of the disease. Such an effort enables resilience, improves emergency preparedness, and empowers quick action to tackle future global health emergencies.

## Data Availability Statement

The original contributions presented in the study are included in the article/supplementary material, further inquiries can be directed to the corresponding author/s.

## Author Contributions

PN, BL, and CWit contributed to the conception of the study and initiated the writing of the manuscript. MS, SL, JS, CK, KS, SE, LV, and CWil contributed to crucial discussions and development of the study project, and actively participated in the writing of the manuscript. All authors contributed to the article and approved the submitted version.

## Conflict of Interest

The authors declare that the research was conducted in the absence of any commercial or financial relationships that could be construed as a potential conflict of interest.
